# Risk factors and outcomes of patients with ocular involvement of candidemia

**DOI:** 10.1371/journal.pone.0222356

**Published:** 2019-09-06

**Authors:** Hyo-Ju Son, Min Jae Kim, Suhwan Lee, Sungim Choi, Kyung Hwa Jung, Jiwon Jung, Yong Pil Chong, Sung-Han Kim, Sang-Ho Choi, Yang Soo Kim, Jun Hee Woo, Joo Yong Lee, Sang-Oh Lee

**Affiliations:** 1 Department of Infectious Diseases, Asan Medical Center, University of Ulsan College of Medicine, Seoul, South Korea; 2 Department of Ophthalmology, Kangwon National University Hospital, Kangwon National University Graduate School of Medicine, Chuncheon, Korea; 3 Department of Ophthalmology, Asan Medical Center, University of Ulsan College of Medicine, Seoul, South Korea; University of Pittsburgh, UNITED STATES

## Abstract

**Background:**

Ocular involvement of candidemia can result in serious complications, including vision loss. This study investigated the risk factors for ocular involvement in patients with candidemia and the outcomes of treatment.

**Methods:**

Episodes of candidemia in hospitalized adults who underwent ophthalmic examinations within 2 weeks of candidemia onset between January 2014 and May 2017 were retrospectively reviewed. Their demographic characteristics, antifungal treatments, and visual outcomes were evaluated.

**Results:**

During the study period, 438 adults were diagnosed with candidemia, with 275 (62.8%) undergoing ophthalmic examinations within 2 weeks. Of these 275 patients, 59 (21.5%) had fundoscopic abnormalities suggestive of ocular involvement, including 51 with chorioretinitis and eight with *Candida* endophthalmitis. Eleven patients were symptomatic. Persistent candidemia (adjusted odd ratio [aOR], 2.55; 95% confidence interval [CI], 1.29–5.08; *P* = 0.01), neutropenia during the preceding 2 weeks (aOR, 2.92; 95% CI, 1.14–7.53; *P* = 0.03), and *C*. *albicans* infection (aOR, 2.15; 95% CI, 1.09–4.24; *P* = 0.03) were independently associated with ocular involvement. Among the 24 patients with neutropenia, 41.7% had ocular involvements at the initial examination. Ophthalmologic examination even before the neutrophil recovery was positive in one-third of neutropenic patients. Out of the 37 patients in whom ocular outcomes after 6 weeks were available, 35 patients showed favorable or stable fundoscopic findings. Two patients had decreased visual acuity despite the stable fundoscopic finding.

**Conclusion:**

Neutropenia within two weeks of candidemia was a risk factor for ocular involvement. More than 80 percent of patients with ocular involvements were asymptomatic, emphasizing the importance of routine ophthalmic examinations. The median 6 weeks of systemic antifungal treatment resulted in favorable outcomes in 89.2% of patients.

## Introduction

Candidemia is a common cause of bloodstream infection worldwide and is frequently associated with significant morbidity and mortality rates [1]. *Candida* can infect nearly every organ in the body and many types of prosthetic materials, with the female genital tract, oral mucosa, kidney, brain, heart, and eyes being the sites most commonly affected [2]. Endophthalmitis is a rare but severe form of ocular inflammation caused by infection of the intraocular cavity, and can lead to irreversible visual loss if not treated properly and promptly [3]. Rates of *Candida* endophthalmitis have been reported to range from 0% to 1.6% [4–7], and rates of total ocular involvement from 2.7% to 37% [4–9]. Fundoscopic examination is important in patients with candidemia, because patients with ocular involvement require prolonged antibiotic therapy, and often necessitate adjuvant therapy such as intravitreal treatment or surgery. Although these patients are usually treated with systemic antifungal agents for 4–6 weeks, few studies to date have assessed treatment outcomes in candidemia patients with ocular involvement [4–9]. This study assessed the frequency of ocular involvement and treatment outcomes in patients with candidemia, as well as risk factors for ocular involvement.

## Materials and methods

### Setting, patients, and study design

All episodes of candidemia in adult patients hospitalized at Asan Medical Center, a 2,700-bed tertiary referral center in Seoul, South Korea, between January 2014 and May 2017 were retrospectively evaluated, and those patients who underwent ophthalmic examinations within 2 weeks of candidemia onset were selected. Fundoscopic findings were reviewed, and ocular infections were categorized as endophthalmitis or chorioretinitis and further classified as ‘proven’, ‘probable’, or ‘possible’ by a retinal specialist [6, 7].

### General definitions

Candidemia was defined as the presence of at least one blood culture positive for *Candida* species. If patients had more than one episode of candidemia, only the first episode was analyzed. Neutropenia was defined as an absolute neutrophil count <500 cells/μL. Any duration of neutropenia within 2 weeks prior to candidemia onset was counted. Corticosteroid therapy was defined as receiving systemic glucocorticosteroids for any reason in the past six weeks, regardless of dose [10]. Persistent candidemia was defined as persistently positive blood cultures ≥72 hours following treatment initiation [11].

### Definition of ocular involvement of candidemia

Proven ocular infection was defined as ocular lesions in combination with positive culture of vitreous aspirates. Probable endophthalmitis was defined as vitritis or fluffy lesions with extension into the vitreous. Probable chorioretinitis was defined as deep focal white infiltrates in the retina. In addition, hemorrhage, Roth spots, and nerve fiber layer infarctions (cotton wool spots) in candidemia patients were classified as probable chorioretinitis if no other cause for these abnormalities (e.g., diabetes mellitus, hypertension, or thrombocytopenia) could be identified. Possible chorioretinitis was defined as signs of chorioretinitis observed in patients with underlying systemic diseases that may cause similar lesions (e.g., diabetic or hypertensive retinopathy, thrombocytopenia, or cancer metastasis) [6, 7].

### Statistical analysis

Categorical variables were compared using the χ^2^ or Fisher’s exact test, as appropriate, and continuous variables were compared using Student’s t-test or the Mann-Whitney U-test, as appropriate. All tests of significance were two-tailed, and *P* values <0.05 were considered statistically significant. Risk factors for ocular involvement were analyzed by backward stepwise logistic regression analysis. All variables significant in the univariate analysis and other variables of clinical importance were included in a multiple logistic regression model. All statistical analyses were performed using SPSS for Windows software package, version 24 (SPSS Inc., Chicago, IL, USA).

### Ethical approval

This observational study was approved by the institutional review board of the Asan Medical Center (IRB No. 2017–0794). To protect personal privacy, identifying information in the electronic database was encrypted. Informed consent was waived by the ethics committee because no intervention was involved and no patient-identifying information was included.

## Results

### Comparison of characteristics of patients according to ophthalmic examination

Of the 438 patients diagnosed with candidemia during the study period, 163 (37.2%) did not undergo fundoscopic examination within 2 weeks of candidemia onset. Patients with persistent candidemia were more likely to undergo ophthalmic examinations compared with those without (20.4% versus 8.3%; *P* = 0.002). Compared with patients who underwent ophthalmologic examination, those who did not had higher likelihood of septic shock state (16.0% versus 35.0%; *P*<0.001), early mortality (2.5% versus 28.8%; *P*<0.001), and 30-day mortality (17.8% versus 48.8%; *P*<0.001). (Table 1).

**Table 1:** Demographic factors, clinical manifestations, and outcomes in patients according to ophthalmic examination within 2 weeks of candidemia.

Characteristic or outcome	Patients who underwent ophthalmic exam (n = 275)	Patients who did not undergo ophthalmic exam (n = 163)	*P*
Demographics			
**Male sex**	**179 (65.1)**	**87 (53.4)**	**0.02**
Age (mean±SD) (years)	63.2±13.8	61.2±13.9	0.91
Comorbid conditions			
Malignancy			
solid organ	138 (50.2)	83 (51.2)	0.83
hematologic	32 (11.7)	28 (17.2)	0.11
Diabetes mellitus	75 (27.3)	51 (31.3)	0.37
Heart failure	33 (12.0)	17 (10.4)	0.62
Chronic renal disease	29 (10.5)	15 (9.3)	0.67
Liver cirrhosis	21 (7.6)	21 (12.9)	0.07
Chronic lung disease	14 (5.1)	6 (3.7)	0.49
Risk factors (within 6 weeks)			
Prior antibacterial therapy	261 (94.9)	156 (95.7)	0.71
**Corticosteroid therapy**	**92 (33.5)**	**75 (46.0)**	**0.01**
Prior surgery	88 (32.0)	51 (31.3)	0.88
Chemotherapy	75 (27.3)	55 (33.7)	0.15
Biliary intervention	36 (13.1)	22 (13.5)	0.90
Neutropenia ^a^	24 (8.7)	17 (10.4)	0.67
[median, IQR] (days) ^b^	[8, 5–12]	[9, 5–18]	
Radiosurgery	6 (2.2)	2 (1.2)	0.72
Clinical characteristics (at onset)			
Fever ^c^	196 (71.3)	110 (67.5)	0.40
**ICU admission**	**70 (25.5)**	**67 (41.1)**	**0.001**
**Septic shock**	**44 (16.0)**	**57 (35.0)**	**<0.001**
Portal of entry			
Catheter-related	144 (52.4)	69 (42.3)	0.09
**Intra-abdomen**	**65 (23.6)**	**57 (35.2)**	**0.01**
Primary	39 (14.2)	29 (17.8)	0.32
Urinary tract infection	22 (8.0)	8 (4.9)	0.21
Candida species (no. of isolates)			
*C*. *albicans*	116 (42.2)	54 (33.1)	0.06
*C*. *glabrata*	74 (26.9)	57 (35.0)	0.08
*C*. *tropicalis*	47 (17.1)	28 (17.2)	0.98
*C*. *parapsilosis*	33 (12.0)	14 (8.6)	0.27
*C*. *krusei*	8 (2.9)	6 (3.7)	0.66
Other *Candida* species	6 (2.2)	10 (6.2)	0.03
Outcomes			
**Persistent candidemia**	**56 (20.4)**	**14 (8.3)**	**0.002**
[median, IQR] (days) ^b^	**[8, 6–10]**	**[9, 7–11]**	
**Early mortality (7 days)**	**7 (2.5)**	**47 (28.8)**	**<0.001**
**Death within 30 days**	**49 (17.8)**	**79 (48.8)**	**<0.001**

Data are reported as n (%), except where otherwise indicated.

Abbreviations: SD, standard deviation; HIV, human immunodeficiency virus; IQR, interquartile range; ICU, intensive care unit.

^a^ Within 2 weeks prior to candidemia onset

^b^ The median and interquartile range values were calculated with only the relevant cases.

^c^ Body temperature of the tympanic membrane or axilla at 37.8 °C or higher

### Characteristics of patients with ocular involvements

**Fig 1:**
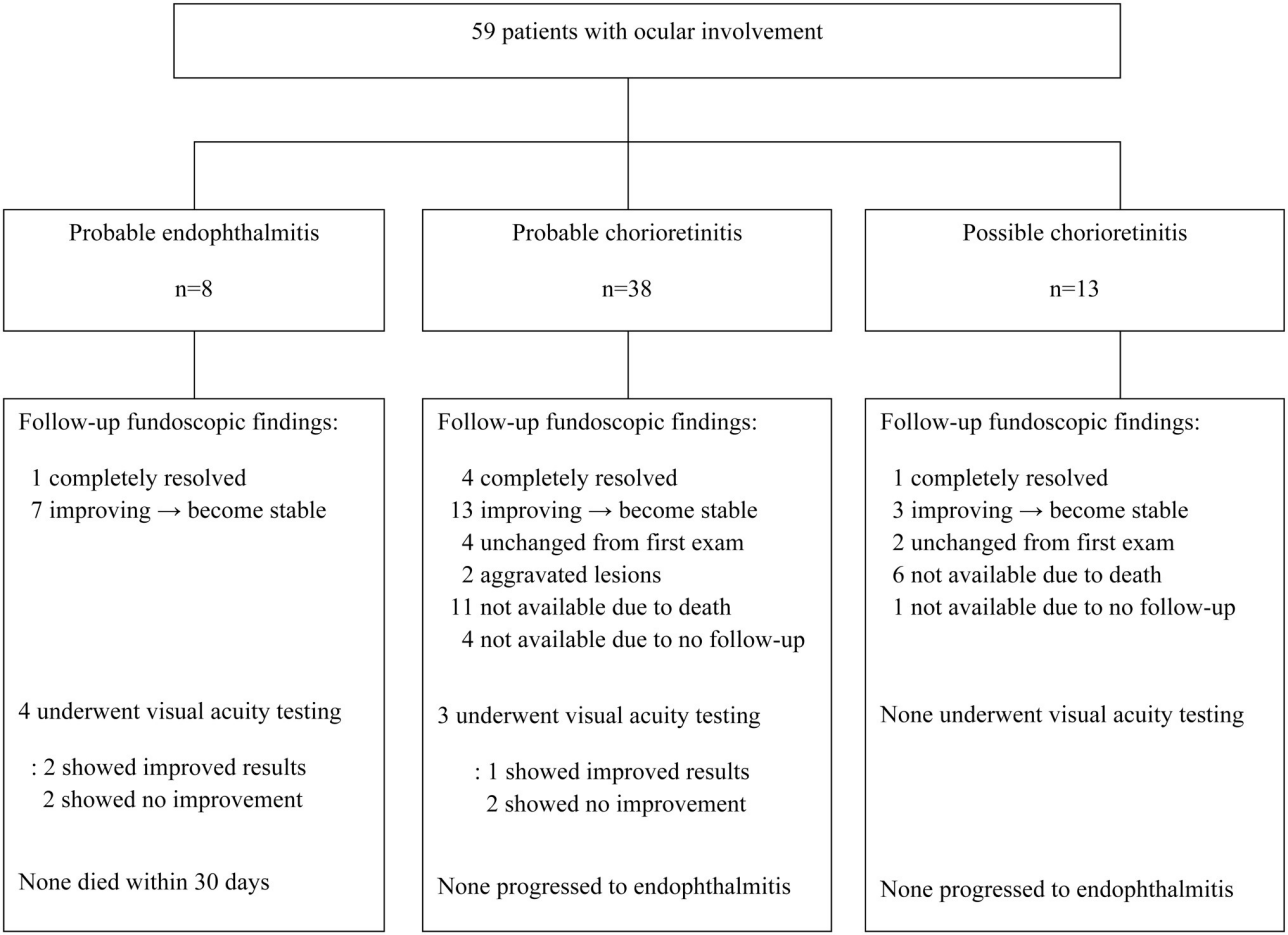
Outcome of ocular manifestations by fundoscopy result and visual acuity.

Of the 438 adult patients diagnosed with candidemia during the study period, 275 (62.8%) underwent ophthalmic examinations within 2 weeks after diagnosis of candidemia. Patients underwent ophthalmic examinations a median 6 days (interquartile range [IQR], 4.0–8.0 days) after candidemia onset. Of the 275 patients who underwent ophthalmic examinations, 59 (21.4%) had ocular involvement, including eight (2.9%) with endophthalmitis and 51 (18.5%) with chorioretinitis. All eight patients with endophthalmitis were classified as having probable disease, because none underwent vitreous sampling (Fig 1). Of the 59 patients with ocular involvement, 47 (79.7%) patients showed involvement in both eyes, nine (15.3%) showed only right eye involvement, and three (5.1%) had only left eye involvement. Of these 59 patients, nine (15.3%) could not describe their subjective visual symptoms due to severe illness or weakened mental state, whereas 11 (18.6%) reported visual disturbances, including four of eight (50%) with endophthalmitis and 7 of 51 (13.7%) with chorioretinitis. Among 11 patients, 9 patients complained of blurred vision, 1 patient had eye floaters and another developed eye ball pain. Although fundoscopic exam was usually performed only once, two patients with initial negative results were later confirmed to have ocular involvements: one had newly developed blurred vision and the other had repeated ophthalmologic examination due to persistent candidemia.

### Risk factors for ocular involvement

Table 2 compares the characteristics of patients with and without ocular involvement. Corticosteroid use during the preceding 6 weeks (45.8% versus 30.1%; *P* = 0.02), chemotherapy during the preceding 6 weeks (39.0% versus 24.1%; *P* = 0.02), neutropenia during the preceding 2 weeks (16.9% versus 6.5%; *P* = 0.02), *C*. *albicans* infection (61.0% versus 37.0%; *P* = 0.001), and persistent candidemia (33.9% versus 16.7%; *P* = 0.004) were significantly more frequent among patients with than without ocular involvement. By contrast, *C*. *glabrata* infection (8.5% versus 31.9%; *P*<0.000) was significantly less frequent among patients with than without ocular involvement. However, underlying diseases, presumed sources of candidemia, types of antifungal treatment before ophthalmologic examination and clinical characteristics at candidemia onset did not differ significantly in these two groups (Table 2). Multivariate analysis of factors with *P* values <0.05 on univariate analysis found that neutropenia during the preceding 2 weeks (adjusted odds ratio [aOR], 2.92; 95% confidence interval [CI], 1.14–7.53; *P* = 0.03), *C*. *albicans* infection (aOR, 2.15; 95% CI, 1.09–4.24; *P* = 0.03), and persistent candidemia (aOR, 2.55; 95% CI, 1.29–5.08; *P* = 0.01) were independently associated with ocular involvement, whereas *C*. *glabrata* infection (aOR, 0.32; 95% CI, 0.11–0.91; *P* = 0.03) was significantly but inversely associated with ocular involvement (Table 3).

**Table 2:** Demographic characteristics, clinical manifestations, and outcomes in candidemia patients with and without ocular involvement.

Characteristics or outcome	Patients with ocular involvement (n = 59)	Patients without ocular involvement (n = 216)	*P*
Demographics			
Male sex	36 (61.0)	143 (66.2)	0.46
Age (mean±SD) (years)	61.0±13.7	63.7±13.9	0.19
Comorbid conditions			
Malignancy			
solid organ	28 (47.5)	110 (50.9)	0.64
hematologic	8 (13.6)	24 (11.2)	0.61
Diabetes mellitus	12 (20.3)	63 (29.2)	0.18
Chronic renal disease	8 (13.6)	21 (9.7)	0.40
Heart failure	5 (8.5)	28 (13.0)	0.35
Liver cirrhosis	3 (5.1)	18 (8.3)	0.58
Chronic lung disease	3 (5.1)	11 (5.1)	1.00
HIV infection	0 (0.0)	1 (0.5)	1.00
Risk factors (within 6 weeks)			
Prior antibacterial therapy	53 (89.8)	208 (96.3)	0.05
**Corticosteroid therapy**	**27 (45.8)**	**65 (30.1)**	**0.02**
**Chemotherapy**	**23 (39.0)**	**52 (24.1)**	**0.02**
Prior surgery	18 (30.5)	70 (32.4)	0.78
**Neutropenia**^a^	**10 (16.9)**	**14 (6.5)**	**0.02**
[median, IQR] (days)^b^	[8, 5–11]	[8, 5–13]	
Immunosuppressive therapy	7 (11.9)	16 (7.4)	0.27
Biliary intervention	6 (10.2)	30 (13.9)	0.45
Radiosurgery	2 (3.4)	4 (1.9)	0.61
Clinical characteristics (at onset)			
Fever^c^	45 (76.3)	151 (69.9)	0.34
ICU requirement	10 (16.9)	60 (27.9)	0.09
Septic shock	8 (13.6)	36 (16.7)	0.56
Portal of entry			
Catheter related	30 (50.8)	114 (52.8)	0.79
Intra-abdominal	16 (27.1)	49 (22.7)	0.48
Primary	8 (13.6)	31 (14.4)	0.88
Urinary tract infection	3 (5.1)	19 (8.8)	0.43
*Candida* species (no. of isolates)			
***C*. *albicans***	**36 (61.0)**	**80 (37.0)**	**0.001**
*C*. *tropicalis*	13 (22.0)	34 (15.7)	0.26
***C*. *glabrata***	**5 (8.5)**	**69 (31.9)**	**0.000**
*C*. *parapsilosis*	3 (5.1)	30 (13.9)	0.07
*C*. *krusei*	2 (3.4)	6 (2.8)	0.68
Other *Candida* species	1 (1.7)	5 (2.3)	1.00
Antifungal treatment^d^			
Fluconazole	42 (71.2)	126 (58.3)	0.10
Echinocandin	13 (22.0)	77 (35.6)	0.07
Amphotericin	4 (6.8)	11 (5.1)	0.54
Voriconazole	0 (0.0)	2 (0.9)	0.99
Outcomes			
**Persistent candidemia**	**20 (33.9)**	**36 (16.7)**	**0.004**
[median, IQR] (days)^b^	**[8, 4–13]**	**[7, 6–9]**	
Early mortality (7 days)	2 (3.34)	5 (2.3)	0.64
Death within 30 days	13 (22.0)	36 (16.7)	0.34

Data are reported as n (%), except where otherwise indicated.

Abbreviations: SD, standard deviation; HIV, human immunodeficiency virus; IQR, interquartile range; ICU, intensive care unit.

^a^ Within 2 weeks prior to candidemia onset

^b^ The median and interquartile range values were calculated with only the relevant cases.

^c^ Body temperature of the tympanic membrane or axilla at 37.8 °C or higher

^d^ Antifungal treatment for candidemia before ophthalmologic examination

**Table 3:** Univariate and multivariate analyses of risk factors for ocular infections among patients with candidemia (n = 275).

Variable	Univariate analysis OR (95% CI)	Multivariate analysis	
		aOR (95% CI)	*P*
Chemotherapy	2.02 (1.10–3.71)		
Corticosteroid therapy	1.96 (1.09–3.53)		
Neutropenia	2.95 (1.23–7.03)	2.92 (1.14–7.53)	0.03
*C*. *albicans*	2.66 (1.47–4.81)	2.15 (1.09–4.24)	0.03
*C*. *glabrata*	0.20 (0.08–0.52)	0.32 (0.11–0.91)	0.03
Persistent candidemia	2.56 (1.34–4.90)	2.55 (1.29–5.08)	0.01

Abbreviations: aOR, adjusted odds ratio; OR, odds ratio; CI, confidence interval.

Regarding 24 patients with neutropenia within 2 weeks, 12 patients were neutropenic and the other 12 recovered from neutropenia at the time of candidemia. Four among the 12 neutropenic patients at the time of candidemia showed ocular involvement at initial examination; two of these patients developed more prominent ocular lesions as neutrophil count increased, and two died before neutrophil recovery. Of the eight neutropenic patients with normal fundoscopic results at first examination, only two underwent an additional ophthalmologic examination without any abnormal lesions. Three patients did not undergo subsequent ophthalmologic examination and the other three died before neutrophil recovery. Among the other 12 patients who recovered from neutropenia at the time of candidemia, 6 had ocular involvements at the initial examination. No further ophthalmologic examination was performed for these patients.

### Treatment outcomes of ocular infection

Fundoscopic results 6 weeks after treatment were available for 37 (62.7%) of the 59 patients with ocular involvement. Of the other 22 patients, 17 died before outcomes were assessed and five did not undergo follow-up examinations. The 37 patients included eight with probable endophthalmitis, 23 with probable chorioretinitis, and six with possible chorioretinitis. The causative organisms were *C*. *albicans* in 22 patients, *C*. *tropicalis* in eight, *C*. *parapsilosis* in three, *C*. *glabrata* in three, and *C*. *guilliermondii* in one. Of these 37 patients, 34 (91.9%) were treated with fluconazole, and 10 (2.7%) received intravitreal treatment. Follow-up examinations at 6 weeks revealed improved lesions in 29 (78.5%) of the 37 patients, including all eight (100%) with probable endophthalmitis, 17 (73.9%) of 22 with probable chorioretinitis, and four (66.7%) of six with possible chorioretinitis. The ocular lesions in four (17.4%) patients with probable chorioretinitis and two (33.3%) with possible chorioretinitis were similar to the lesions of initial fundoscopic examinations. Ocular lesions worsened in two patients, resulting in vitrectomy (Table 4). Unfortunately, visual acuity results were available only in seven patients, four with endophthalmitis and three with chorioretinitis. Two with endophthalmitis and another two with chorioretinitis did not show full recovery of visual acuity (S1 Table).

**Table 4:** Treatment of patients with ocular involvement.

Age/Sex	Candida Species	Anti-fungal Agent	Intravitreal Injection	Vitrectomy	Treatment Duration	Follow-up fundoscopic findings
Probable Endophthalmitis						
68/M	*C*. *tropicalis*	Fluconazole	Yes	No	56 Days	Improved
62/M	*C*. *albicans*	Fluconazole	Yes	No	55 Days	Improved
73/F	*C*. *tropicalis*	Fluconazole	Yes	No	61 Days	Improved
65/M	*C*. *albicans*	Fluconazole	Yes	No	48 Days	Improved
37/M	*C*. *tropicalis*	Fluconazole, + Amphotericin B	Yes	No	70 Days	Improved
49/M	*C*. *parapsilosis*	Fluconazole	No	No	28 Days	Resolved
38/F	*C*. *albicans*	Fluconazole	Yes	No	60 Days	Improved
59/M	*C*. *albicans*	Fluconazole	No	No	30 Days	Improved
Probable Chorioretinitis						
63/M	*C*. *tropicalis*	Fluconazole	No	No	42 Days	Resolved
91/F	*C*. *albicans*	Fluconazole	No	No	20 Days	Improved
91/M	*C*. *albicans*	Fluconazole	No	No	56 Days	Improved
66/M	*C*. *tropicalis*	Fluconazole	No	No	58 Days	Improved
62/M	*C*. *albicans*	Fluconazole, + Caspofungin	Yes	No	73 Days	Improved
79/F	*C*. *albicans*	Fluconazole	No	No	50 Days	Improved
77/M	*C*. *albicans*	Fluconazole	No	No	18 Days	Improved
**35/M**	***C*. *albicans***	**Fluconazole**	**Yes**	**Yes**	**89 Days**	**Aggravated**
62/M	*C*. *albicans*	Fluconazole	No	No	40 Days	Resolved
74/F	*C*. *albicans*	Fluconazole	No	No	24 Days	Resolved
71/M	*C*. *albicans*	Fluconazole	No	No	39 Days	Stable
77/M	*C*. *albicans*	Fluconazole	No	No	52 Days	Improved
70/M	*C*. *albicans*	Fluconazole	No	No	18 Days	Stable
75/F	*C*. *tropicalis*	Fluconazole	No	No	25 Days	Improved
**32/F**	***C*. *tropicalis***	**Fluconazole**	**Yes**	**Yes**	**97 Days**	**Aggravated**
55/M	*C*. *parapsilosis*	Amphotericin B	No	No	28 Days	Improved
61/F	*C*. *albicans*	Fluconazole	No	No	42 Days	Resolved
57/M	*C*. *parapsilosis*	Fluconazole	No	No	27 Days	Improved
33/M	*C*. *albicans*	Fluconazole	No	No	42 Days	Improved
45/F	*C*. *albicans*	Fluconazole	No	No	60 Days	Improved
62/F	*C*. *albicans*	Fluconazole	No	No	42 Days	Improved
77/M	*C*. *albicans*	Fluconazole	No	No	28 Days	Stable
39/F	*C*. *albicans*	Fluconazole	No	No	28 Days	Stable
81/M	*C*. *tropicalis*	Anidulofungin	No	No	21 Days	Died
68/F	*C*. *albicans*	Fluconazole	No	No	25 Days	Died
64/F	*C*. *krusei*	Amphotericin B	No	No	34 Days	Died
55/M	*C*. *albicans*	Fluconazole	No	No	9 Days	No follow-up
46/F	*C*. *albicans*	Fluconazole	No	No	15 Days	Died
43/F	*C*. *albicans*	Fluconazole	Yes	No	40 Days	No follow-up
70/M	*C*. *albicans*	Fluconazole	No	No	19 Days	Died
53/F	*C*. *tropicalis*	Micafungin	No	No	7 Days	No follow-up
76/M	*C*. *albicans*	Fluconazole	No	No	13 Days	No follow-up
51/M	*C*. *albicans*	Fluconazole	No	No	8 Days	Died
61/M	*C*. *albicans*	Fluconazole	No	No	10 Days	Died
65/M	*C*. *tropicalis*	Anidulofungin	No	No	15 Days	Died
49/F	*C*. *albicans*	Fluconazole	No	No	7 Days	Died
61/F	*C*. *albicans*	Amphotericin B	No	No	27 Days	Died
58/M	*C*. *albicans*	Anidulofungin	No	No	21 Days	Died
Possible Chorioretinitis						
61/F	*C*. *glabrata*	Micafungin	No	No	14 Days	Improved
49/M	*C*. *glabrata*	Fluconazole	No	No	35 Days	Improved
65/M	*C*. *glabrata*	Caspofungin	No	No	16 Days	Resolved
67/F	*C*. *guilliermondii*	Fluconazole	No	No	26 Days	Stable
77/F	*C*. *tropicalis*	Fluconazole	No	No	21 Days	Improved
45/M	*C*. *albicans*	Fluconazole	No	No	56 Days	Stable
65/M	*C*. *glabrata*	Micafungin	No	No	40 Days	Died
72/F	*C*. *albicans*	Amphotericin B	No	No	26 Days	Died
63/F	*C*. *albicans*	Fluconazole	No	No	10 Days	Died
55/M	*C*. *tropicalis*	Voriconazole	No	No	23 Days	Died
45/M	*C*. *krusei*	Caspofungin	No	No	18 Days	No follow-up
66/M	*C*. *albicans*	Anidulofungin	No	No	27 Days	Died
66/M	*C*. *tropicalis*	Amphotericin B	No	No	28 Days	Died

### Detailed description of the patients that worsened during treatment

Two patients had poor ophthalmologic outcomes. One patient was a 32-year-old woman with acute myeloid leukemia who was in a neutropenic state after cytotoxic chemotherapy and was diagnosed with *C*. *tropicalis* candidemia. She started to receive caspofungin on the day of yeast detection in the blood and fluconazole was added in the following two days. Examination by an ophthalmologist three days after candidemia onset revealed chorioretinitis in both eyes. She received caspofungin alone from the fourth day to the twelfth day after candidemia onset and changed to fluconazole from the thirteenth day. When her eyes were examined three days after the first examination, the chorioretinitis in her right eye had improved but the chorioretinitis in her left eye had been exacerbated during the echinocandin treatment and more aggravated with neutrophil recovery despite systemic fluconazole and intravitreal voriconazole injection. Vitreal hemorrhage occurred, and although the patient underwent vitrectomy, vision was not recovered.

The other patient was a 35-year-old man with Crohn’s disease who required long-term parenteral feeding and was diagnosed with central venous catheter-related C. albicans candidemia. He started to receive fluconazole upon detection of yeast in the blood culture and continued to receive fluconazole thereafter. Examination by an ophthalmologist four days after candidemia onset showed chorioretinitis in both eyes. The chorioretinitis in his right eye had improved, but the subfoveal infiltration in his left eye had aggravated during treatment with systemic fluconazole and intravitreal voriconazole injection. He finally underwent vitrectomy of the left eye due to the new epiretinal membrane.

## Discussion

To our knowledge, few studies have analyzed risk factors and treatment outcomes of ocular involvement in patients with candidemia [4–9]. Of the 275 patients with candidemia who underwent fundus examination during the study period, 59 (21.5%) had fundoscopic abnormalities suggesting ocular involvement of *Candida*. Fungemia with *C*. *albicans*, persistent candidemia, and neutropenia during the preceding 2 weeks were factors independently associated with ocular involvement, whereas *C*. *glabrata* was inversely associated with ocular manifestations. Outcomes after 6 weeks of treatment were available for 37 patients, including 34 treated with fluconazole for a median 42 days (IQR, 27–56 days) and 10 who required intravitreal treatment. Fundoscopic findings improved or stabilized after 6 weeks in all but two patients, both of whom underwent vitrectomy. Two patients had decreased visual acuity despite the stable fundoscopic finding. Collectively speaking, we found that 6 weeks of systemic antifungal treatment contributed to good outcomes in 89.2% of patients.

The rates of ocular involvement of candidemia have been reported to vary, from 0% to 1.6% for endophthalmitis and 2.7% to 37.0% for any ocular involvement [4–9]. In the present study, 59 of 275 (21.5%) patients had fundoscopic abnormalities suggestive of *Candida* eye involvement. These 59 patients included eight with probable endophthalmitis, 38 with probable chorioretinitis, and 13 with possible chorioretinitis. However, this prevalence may have been underestimated due to selection bias. Of the 438 patients diagnosed with candidemia during the study period, 163 (37.2%) did not undergo fundoscopic examination within 2 weeks of candidemia onset. These patients were significantly more likely to have septic shock and to have a significantly higher mortality rate. Thus this study does not properly reflect the prevalence of ocular involvement of candidemia in patients with more severe disease, who also have many risk factors for ocular involvement.

Our findings support the notion that ocular candidiasis is often asymptomatic [7]. Of the 59 patients with ocular involvement, nine could not describe their subjective visual symptoms due to severe illness or weakened mental state. Only eleven (18.6%) reported visual disturbances, including four of eight (50%) with endophthalmitis and 7 of 51 (13.7%) with chorioretinitis. Blurred vision was the most common symptom, followed by eye pain and eye floaters. Forty (67.8%) had no ocular symptoms in the beginning, and one developed blurred vision during treatment. Candidemia seeds the highly vascular choroid first, so the initial manifestation is usually chorioretinitis or choroiditis [12]. Initially, there may be minimal or no vitreous inflammation. Infection of the choroid and retina often does not cause pain; therefore, unless the lesions are near the macula, patients with early *Candida* chorioretinitis may be asymptomatic. As the infection worsens, vitritis develops and vision becomes impaired [12]. Therefore, routine fundoscopy to detect ocular candidiasis is recommended, even in patients with candidemia who do not present with visual symptoms. In our study, patients with endophthalmitis were likely to be symptomatic than those with chorioretinitis. However, half of the patients with endophthalmitis did not complain of any visual problems, emphasizing the importance of routine ocular examination.

Risk factors for endogenous *Candida* chorioretinitis and endophthalmitis are poorly understood, although studies suggest that these risk factors are similar to those for candidemia itself [13, 14]. Fungemia with *C*. *albicans* (versus non-albicans species), persistent candidemia, visual symptoms, immunosuppression, and central catheter placement have been associated with ocular candidiasis [5, 7, 15]. The present study found that fungemia with *C*. *albicans*, persistent candidemia, and neutropenia during the preceding 2 weeks were independently associated with ocular involvement. Similar to previous studies, we found that *C*. *albicans* was the most common cause of ocular candidiasis, followed by *C*. *tropicalis* [7, 16], with *C*. *glabrata* infection being significantly less frequent in patients with than without ocular manifestations.

In general, fundoscopic examinations are less sensitive in neutropenic than in non-neutropenic patients [17]. In the present study, we found that four patients, representing one-third of neutropenic patients at the time of candidemia, had ocular involvements. Among them, two patients developed more prominent lesions with neutrophil recovery. Within our limited data, no patients without initial ocular involvements revealed newly developing ocular lesions with neutrophil recovery. Current guidelines recommend performing ophthalmologic examination after neutrophil recovery and echinocandins as initial antifungal therapy for candidemia [17]. Although fundoscopy at the time of neutropenia is relatively insensitive, our data suggests routine ocular examination at the time of neutropenia could also be helpful. Further research is required for optimal timing of sequential fundoscopic examinations and to empirically evaluate antifungal agents administered to patients at high risk for ocular involvement.

Although echinocandin is currently recommended as the first-line antifungal agent for candidemia, there is a growing concern that the use of echinocandin as an initial antifungal therapy may increase the risk of ocular invasion compared with triazoles. The penetration of echinocandins into the eye has been shown to be poor in previous studies [18–20]. However, in the present study, initial echinocandin therapy was not associated with the rate of ocular involvement. Although it can be assumed that echinocandins may reduce the duration of candidemia and counterbalance its negative pharmacologic effect, this assumption could not be verified with our data. Considering that existing data on the rate and outcome of ocular candidiasis in patients initially treated with echinocandins are scarce and conflicting [20–22], further studies need to be carried out to directly address this issue.

Fundoscopic findings improved or stabilized within 6 weeks in 35 of the 37 patients for whom 6 week outcomes were available. Thirty-two patients with favorable fundoscopic outcomes were treated with fluconazole for a median 42 days (IQR, 27–56 days). However, despite favorable fundoscopic outcome, two with endophthalmitis and another two with chorioretinitis did not show full recovery of visual acuity. This shows that 10.8% of patients have vision deterioration. Active treatment strategy including intravenous and intravitreal therapy should be considered because visual acuity reflects the functional outcomes of the eye.

It is recommended that patients with *Candida* endophthalmitis be treated for at least 4–6 weeks, with the final duration depending on resolution of the lesions [17]. The patients in the present study were treated for a median 6 weeks, with 89.2% experiencing successful outcomes. A total of 18 out of 37 patients (40.5%) were treated for less than 6 weeks (median, 26.5 days; IQR, 19.5–28.5 days). All of them showed favorable fundoscopic outcome in 6 weeks after candidemia onset. We suggest that treatment duration can be optimized to 4 to 6 weeks depending on the severity of ocular invasion. These results are in agreement with clinical practice guidelines.

This study had several limitations. First, it was a single-centered retrospective study in South Korea; multicenter prospective studies are needed to confirm our findings. Second, patients with critical illness without bacteremia or candidemia may have retinal lesions similar to those described here as chorioretinitis [23]. However, differentiating between infectious chorioretinitis and retinal lesions of noninfectious causes is difficult. Thus there could be diagnostic uncertainty of possible cases. However, considering that fundoscopic findings were improved in two-thirds of possible chorioretinitis patients after anti-fungal treatment, we believe that most of the possible cases truly originated from candidemia and thus do not significantly weaken our findings. Third, although patients may develop new ocular lesions after the start of antifungal treatment [11], periodic follow-up ophthalmic examinations were not performed in patients without baseline abnormalities unless they complained of vision symptoms. Thus, patients with late onset ocular candidiasis may have been omitted.

In conclusion, ocular involvement is common in patients with candidemia and is associated with persistent candidemia, fungemia with *C*. *albicans*, and neutropenia during the 2 weeks prior to candidemia. Ophthalmologic examination even before neutrophil recovery was positive in one-third of neutropenic patients. More than 80 percent of patients with ocular involvements were asymptomatic, emphasizing the importance of routine ophthalmic examinations. A median 6 weeks of systemic antifungal treatment resulted in favorable outcome in 89.2% of patients, in agreement with current guidelines.

## Supporting information

S1 Table Visual acuity results of seven patients with ocular involvement of candidemia. Abbreviations: VA, visual acuity; OD, right eye; OS, left eye. *: Time after the treatment initiation.
(DOCX)
