# Supplementary material for: Risk factors and outcomes of patients with ocular involvement of candidemia
Source: PLoS One. 2019 Sep 6;14(9):e0222356. doi: 10.1371/journal.pone.0222356 (PMC6730936; doi:10.1371/journal.pone.0222356)
Supplement: S1 Table — Abbreviations: VA, visual acuity; OD, right eye; OS, left eye. *: Time after the treatment initiation. (DOCX) [file pone.0222356.s001.docx]

**S1 Table. Visual acuity results of seven patients with ocular involvement of candidemia**

|  | Initial VA  Results  (OD OS) | VA during the treatment  (OD OS, *) | VA at 6 weeks  (OD OS) | Last VA results  (OD OS, *) | VA recovery  after treatment |
| --- | --- | --- | --- | --- | --- |
| Patient 1 | (20/25 20/50) | (20/20 20/200, 2 weeks) | (20/20 20/320) | (20/20 20/320, 8 weeks) | No |
| Patient 2 | (20/800 20/320) | (20/800 20/200, 2 weeks) | (20/2000 20/200) | (20/2000 20/200, 6 weeks) | No |
| Patient 3 | (20/16 20/16) | (20/80 20/25, 1 week) | (20/50 20/100) | (20/20 20/200, 15 weeks) | No |
| Patient 4 | (20/20 20/50) | (20/20 20/320, 1 week) | (20/16 20/125) | (20/16 20/320, 16 weeks) | No |
| Patient 5 | (20/32 20/50) | (20/25 20/125, 2 weeks) | (20/25 20/63) | (20/32 20/63, 15 weeks) | Yes |
| Patient 6 | (20/32 20/32) | (20/80 20/40, 1 week) | (20/32 20/32) | (20/32 20/32, 6 weeks) | Yes |
| Patient 7 | (20/63 20/100) | (20/40 20/63, 1 week) | (20/40 20/50) | (20/40 20/50, 6 weeks) | Yes |

Abbreviations: VA, visual acuity; OD, right eye; OS, left eye

* : Time after the treatment initiation
